# Biological and toxicological evaluation of chicken eggshell membrane for guided bone regeneration: An *in vitro* assessment

**DOI:** 10.6026/973206300221915

**Published:** 2026-03-31

**Authors:** Deepak Chandrasekaran, Sharada T Rajan, Ravindran Chinnaswami, Nadathur Doraiswamy Jayakumar

**Affiliations:** 1 Department of Oral and Maxillofacial Surgery, Sri Ramachandra Dental College and Hospital, SRIHER, Chennai, India; 2 Department of Oral & Maxillofacial Pathology, Sri Ramachandra Dental College & Hospital, SRIHER, Chennai, India; 3 Department of Periodontics & Implantology, Saveetha Dental College, Chennai, India

**Keywords:** Guided bone regeneration (GBR), eggshell membrane, barrier membrane, biocompatibility, toxicological evaluation, biomaterials, collagen-based membrane, bone tissue engineering

## Abstract

The lack of cost-effective, biocompatible and sustainable barrier membranes remains a significant limitation in guided bone regeneration (GBR). Therefore, it is of interest to evaluate the biological compatibility and toxicological safety of chicken eggshell membrane as a potential GBR barrier membrane using standardized *in vitro* assays. Physical characterization, cell viability, proliferation, cytotoxicity grading and toxicological assessments were performed under controlled laboratory conditions. The membrane demonstrated high cell viability (>94%), progressive proliferation, non-cytotoxic classification and absence of inflammatory or necrotic responses. Thus, we show that chicken eggshell membrane exhibits excellent biocompatibility with minimal toxicological risk, supporting its potential as a sustainable alternative membrane for GBR applications.

## Background:

Guided bone regeneration (GBR) is a predictable surgical technique used to promote osteogenesis by excluding soft tissue infiltration through barrier membrane placement [1]. Successful GBR requires membranes with adequate mechanical stability, controlled porosity, biocompatibility and appropriate degradation kinetics [2]. Conventional resorbable and non-resorbable membranes have demonstrated clinical efficacy but are associated with limitations including high cost, inflammatory reactions, secondary surgical removal and inconsistent degradation profiles [3]. These limitations have stimulated interest in alternative biomaterials that are biologically safe, structurally adequate and economically sustainable [4]. Naturally derived collagen-based membranes have gained attention due to their extracellular matrix-like architecture and favorable cellular interactions [5]. Among emerging biomaterials, chicken eggshell membrane has attracted interest because of its collagen-rich composition, fibrous microstructure and intrinsic bioactive components [6]. Recent investigations have demonstrated its capacity to support cell adhesion, proliferation and wound healing responses [7]. Therefore, it is of interest to evaluate the biological compatibility and toxicological safety of chicken eggshell membrane as a potential barrier membrane for guided bone regeneration.

## Materials and Methods:

This experimental *in vitro* study evaluated the biological compatibility and toxicological safety of chicken eggshell membrane as a candidate barrier membrane for guided bone regeneration. Fresh chicken eggshells were collected and mechanically cleaned under aseptic conditions. The inner membranes were carefully separated, washed with sterile distilled water and subjected to standardized decellularization and sterilization protocols. Processed membranes were air-dried and stored under sterile conditions until testing. Physical characterization included measurement of thickness, surface density, porosity and tensile strength using calibrated instruments. Porosity was determined using image-based or gravimetric analysis. Tensile strength was assessed using a universal testing machine under controlled loading conditions. Flexibility was qualitatively evaluated during handling and mechanical testing. Fibroblast-like cells were used for biological assessment and cultured in Dulbecco’s Modified Eagle Medium supplemented with fetal bovine serum and antibiotics. Cells were maintained at 37°C in a humidified 5% CO_2_ atmosphere. Biocompatibility was evaluated using cell viability and proliferation assays at 24, 48 and 72 hours. Cytotoxicity testing was performed using extract-based methods in accordance with ISO standards for biological evaluation of medical devices. Morphological evaluation of cell attachment and spreading on the membrane surface was conducted using microscopic analysis. Toxicological assessment included evaluation of necrotic changes, apoptotic features, membrane blebbing and inflammatory indicators such as cell detachment and swelling. All experiments were performed in triplicate to ensure reproducibility. Data were recorded as mean ± standard deviation. Descriptive statistical analysis was applied to assess cellular response and material safety parameters.

## Results:

The processed chicken eggshell membrane demonstrated structural and mechanical characteristics compatible with barrier membrane applications. The mean thickness was 280 ± 25 µm, with porosity of 62.4 ± 4.8%, indicating adequate permeability for nutrient diffusion. Tensile strength measured 3.9 ± 0.6 MPa, reflecting sufficient mechanical integrity for space maintenance. Surface density averaged 42.6 ± 3.1 g/m^2^ and flexibility was qualitatively assessed as good. Biological evaluation revealed consistently high cell viability following exposure to membrane extracts. Viability values were 94.2 ± 3.5% at 24 hours, 96.8 ± 2.9% at 48 hours and 98.1 ± 2.1% at 72 hours. These values were comparable to the control group (99.3 ± 1.2%), indicating minimal cytotoxic influence. Progressive cell proliferation was observed, with optical density increasing from 0.42 ± 0.05 at 24 hours to 0.83 ± 0.07 at 72 hours. Cytotoxicity assessment demonstrated less than 10% growth inhibition, corresponding to Grade 0 classifications under ISO standards. Microscopic examination confirmed normal spindle-shaped morphology, intact nuclear features and extensive cell spreading. No evidence of vacuolization, membrane blebbing or nuclear fragmentation was observed. Toxicological evaluation showed absence of necrosis, apoptosis, or significant inflammatory indicators. Cell detachment remained below 5% and overall toxic response was classified as negative. Collectively, these findings indicate that chicken eggshell membrane exhibits favorable biological and toxicological performance *in vitro*. Table 1 (see PDF) shows that the processed eggshell membrane exhibited a mean thickness of 280 ± 25 µm, porosity of 62.4 ± 4.8% and tensile strength of 3.9 ± 0.6 MPa, indicating adequate structural integrity and flexibility. Table 2 (see PDF) demonstrates high cell viability exceeding 94% at all evaluated time points, suggesting strong cytocompatibility. Table 3 (see PDF) indicates progressive cell proliferation, with optical density increasing from 0.42 ± 0.05 at 24 hours to 0.83 ± 0.07 at 72 hours. Table 4 (see PDF) shows absence of cell lysis and less than 10% growth inhibition, corresponding to Grade 0 non-cytotoxic classifications. Table 5 (see PDF) demonstrates normal spindle-shaped morphology, intact nuclei and extensive cell spreading following membrane exposure. Table 6 (see PDF) reveals minimal difference in viability between control (99.3 ± 1.2%) and treated groups (97.6 ± 2.4%). Table 7 (see PDF) indicates absence of necrotic or apoptotic features, confirming negative toxicological response. Table 8 (see PDF) demonstrates minimal inflammatory indicators with cell detachment below 5%. Table 9 (see PDF) highlights overall excellent biocompatibility, non-cytotoxicity, favorable cell adhesion and promising suitability for guided bone regeneration.

## Discussion:

This study evaluated the biological compatibility and toxicological safety of chicken eggshell membrane as a candidate barrier membrane for guided bone regeneration. The findings demonstrate favorable structural properties, high cytocompatibility and absence of toxicological risk under *in vitro* conditions [8]. Effective GBR membranes must provide mechanical stability, controlled porosity and biocompatibility to maintain regenerative space while supporting cellular activity [1, 2]. The measured thickness and tensile strength observed in this study fall within ranges reported for naturally derived collagen-based membranes used in regenerative applications [3]. The relatively high porosity may facilitate nutrient diffusion while limiting undesirable soft tissue infiltration, which is critical for successful osteogenesis [9]. Cell viability exceeding 94% across all time points indicates that the processed membrane does not release cytotoxic substances. Progressive proliferation further confirms that the membrane surface supports metabolic activity and cell growth. Similar trends have been reported in recent studies evaluating collagen-rich natural membranes for tissue engineering applications [4, 5]. The non-cytotoxic Grade 0 classification according to ISO standards reinforces the material’s safety profile. Maintenance of normal spindle-shaped morphology and intact nuclear structures indicates preservation of physiological cellular behavior. Toxicological evaluation revealed absence of necrosis, apoptosis and membrane blebbing, suggesting that membrane extracts do not induce cellular stress [10]. Minimal inflammatory indicators and low cell detachment further support its favorable biological response. Excessive inflammatory reactions following membrane placement can compromise regenerative outcomes, making this observation clinically relevant [6]. The intrinsic collagen and glycoprotein composition of eggshell membrane may contribute to enhanced cell adhesion and biocompatibility [7]. Additionally, its natural origin and abundance align with sustainable biomaterial development strategies [8]. Advancement to knowledge in this study lies in the comprehensive integration of physical characterization with standardized cytotoxic and toxicological assessment specifically for GBR application. While eggshell membrane has been explored in wound healing and scaffold research, limited data have systematically evaluated its safety profile in the context of barrier membrane requirements for bone regeneration [11]. By demonstrating mechanical adequacy alongside ISO-compliant biological evaluation, this study strengthens the translational rationale for its consideration in regenerative dentistry. The findings provide structured pre-clinical evidence supporting its development as a sustainable alternative to conventional GBR membranes. Limitations include the exclusive reliance on *in vitro* assessment, which cannot fully replicate *in vivo* immune modulation, enzymatic degradation and biomechanical loading. Future studies should incorporate animal models and controlled clinical evaluation to validate regenerative performance and long-term degradation behavior. Despite these limitations, the consistent biological and toxicological safety demonstrated herein supports further investigation toward clinical application.

## Conclusion:

Chicken eggshell membrane demonstrated favorable mechanical properties, excellent cytocompatibility and absence of toxicological risk under standardized *in vitro* evaluation. The integration of structural characterization with ISO-compliant biological assessment provides strong preclinical evidence supporting its suitability as a barrier membrane for guided bone regeneration. These findings support further *in vivo* validation and position eggshell membrane as a sustainable, low-cost alternative to conventional GBR membranes in regenerative dentistry.
